# Cues for Early Social Skills: Direct Gaze Modulates Newborns' Recognition of Talking Faces

**DOI:** 10.1371/journal.pone.0018610

**Published:** 2011-04-15

**Authors:** Bahia Guellai, Arlette Streri

**Affiliations:** René Descartes University (Paris, France) Laboratory for Psychology of Perception, Unité Mixte de Recherche Centre National de la Recherche Scientifique 8158, Centre Biomédical des Saints-Pères, Paris, France; Royal Holloway, University of London, United Kingdom

## Abstract

Previous studies showed that, from birth, speech and eye gaze are two important cues in guiding early face processing and social cognition. These studies tested the role of each cue independently; however, infants normally perceive speech and eye gaze together. Using a familiarization-test procedure, we first familiarized newborn infants (n = 24) with videos of unfamiliar talking faces with either direct gaze or averted gaze. Newborns were then tested with photographs of the previously seen face and of a new one. The newborns looked longer at the face that previously talked to them, but only in the direct gaze condition. These results highlight the importance of both speech and eye gaze as socio-communicative cues by which infants identify others. They suggest that gaze and infant-directed speech, experienced together, are powerful cues for the development of early social skills.

## Introduction

From birth and throughout life, human beings live in a highly social world and interact almost constantly with each other. Therefore, abilities to perceive and understand social partners and their signals are important aspects of a successful social life. Among the visual stimuli encountered from birth, faces are special as they convey most of the information needed to identify and understand others. When looking at someone's face, a lot can be learned about his or her identity, gender, intentions and emotional states [1], [2], [3], [4]. Moreover, the ability to identify others is a crucial prerequisite for learning about social groups [5], [6]. How does this ability develop from birth, and do the socio-communicative cues conveyed by faces play a role in this process? The present study investigated these questions and tested newborns' ability to identify others in interactive situations, in accord with two powerful social cues: speech and gaze.

Previous developmental studies showed that 3-month-old infants identify others and establish social preferences based on visual cues to gender [7], [8], age [9] and race [10], [11], [12]. Other cues conveyed by faces may also play a role in this process. Two socio-communicative cues seem particularly salient: speech and eye gaze. A line of developmental research showed that soon after birth, and throughout early infancy, young infants prefer listening to infant-directed speech over adult-directed speech [13], [14], [15]. Infant-directed speech is linguistically simplified and characterized by high pitch and exaggerated intonation. Infants also prefer listening to their native language rather than to a foreign language and can also discriminate among different languages based on precise elements such as rhythmic or phonological cues [16], [17], [18], [19], [20], [21]. What about the role of language in guiding young infants' identification of others? Using a visual preference procedure, a previous research [22] showed that American infants as young as 6-month-old looked longer at the video of a woman who previously talked to them in their native language with a native accent, than at a woman who previously spoke in a foreign language (i.e., Spanish). These results suggest that spoken language is a powerful social cue already used by young infants to identify others as potential social partners.

From birth, newborn infants are able to recognize familiar and unfamiliar faces. In studies with presentation of the familiar face (i.e., the mother), newborns systematically prefer looking at their static mother's face as opposed to a stranger one [23], [24], [25], [26], [27]. In studies with unfamiliar faces, newborn infants elicit a novelty preference at test [28], [29], [30] and are able to recognize faces despite changes in viewpoint [31]. The disparity of results between studies with familiar and unfamiliar faces could be explained by the fact that unfamiliar faces are always presented static or in sequential rigid motion [32] whereas in studies with the mother, face-to-face interactions have occurred previously to the test session. During these face-to-face interactions, speech component is an important cue which could modulate face processing. This possibility has been tested in two different experiments. Using a combined preferential looking and head turn procedure, Sai's study [33] observed the importance of previous verbal interactions in guiding newborns' identification of their mother's face. For half of the newborns, their mothers were encouraged to talk to them from birth, while for the other half, mothers were asked not to interact with them verbally. In the test session, seven hours later, all newborns were presented with their mother and another woman side by side. Newborns looked longer at and oriented more to their mother only if she had previously talked to them. Given that foetuses hear their mother's voice and prefer it at birth [34], [35], it is possible that the newborn infants who received verbal interactions were reinforced, and that this reinforcement led to a preference for someone who has been identified as an important social partner. But do gaze and speech also aid infants in identifying other individuals? From birth, newborns encounter many different faces talking to them, so the importance of verbal interactions in face recognition at birth could extend to other faces than the mother's. To test this hypothesis, an experiment has recently presented newborn infants with a familiarization-test procedure with video films of unfamiliar women' faces [36]. Newborns were recruited from a maternity hospital where the majority of the families came from different ethnic origins and spoke different languages. Half of them were familiarized with a woman's face talking to them (Experiment 1), and the other half with a woman's face with lips movements but no speech sounds (Experiment 2). In the test phase, photographs of the familiar face and a new one were shown. Newborns looked longer at the familiar face only in the speech condition. Soon after birth therefore, newborns recognize and show a preference for someone who previously interacted with them verbally. These results suggest that very young infants, tested in naturalistic situations, show preferences for people who have interacted with them [37].

During verbal interaction, another important cue could play a role in guiding newborns' identification of potential social partners: eye gaze. The eye region is an important source of information in social interactions for many different vertebrate species from reptiles to mammals [38]. In humans, contrary to other species, direct gaze sometimes constitutes a positive social signal engaging its target in a social interaction [38]. The social functions of human eye gaze are diverse, including following of someone's gaze to significant objects [39], [40], gathering feedback on the others' reactions and regulating turn-taking in conversation [41], [42], expressing intimacy [43], [44], and inferring mental states [1]. The direction of gaze can also influence our identification, categorization and judgment of others [45]. A behavioral study showed that perceived eye gaze modulates performance in face recognition both at the encoding and retrieval levels, with better performance when facing someone with direct gaze, both in adults and children [46]. The same finding has been observed at 4-months [47]: when presented with photographs of faces, infants were able to recognize a previously seen woman's face, by eliciting a novelty preference, only if the face was first seen with direct rather than averted gaze. These experiments tested the role of eye gaze in face recognition using static faces, whereas in everyday interactions, faces are never seen static: faces talk, laugh, and move. In these more complex situations, other cues such as speech seem to modulate attention to the eye region which may influence face processing. For example, 9-weeks-old infants fixate more an adult's eye region when she is talking to them than when she is looking at them silently [48]. In other words, in face-to-face interactions, eye gaze may not provide with sufficient information to process someone's identity.

Newborn infants are already sensitive to the gaze of others and prefer looking at the photograph of a face with eyes open versus closed [49]. They also prefer looking at a photograph of a face with direct versus averted gaze [50]. These results are consistent with the hypothesis of an innate module devoted to gaze processing [1], [51]. However, these experiments focused only on newborns' sensitivity to the eye region and more precisely to direct gaze using still photographs of faces. The role of direct gaze in face recognition at birth, using interactive situations, has not been tested so far. Nonetheless, it seems that in interactive situations, such as those presented in Coulon et al.'s study [36], direct gaze alone (without verbal interaction) is not a sufficient cue in guiding newborns' identification of previously unfamiliar faces: newborns prefer looking at a woman who previously looked at them and interacted with them verbally, but not a woman who looked at them without speaking. So, speech is an important cue in face recognition at birth. These findings raise a critical question: are speech and direct gaze together necessary for the recognition of unfamiliar faces by newborns, or alternatively, is speech the only effective social cue for newborn infants tested in social situations? The present study addressed this question by testing the role of perceived eye gaze in guiding newborns' identification of talking faces.

## Methods

### Participants

Participants were 24 full-term newborns (14 males) from the maternity hospital of Bichat in Paris. All newborns were in good health (APGAR scores above 9). The mean age was 50.5 hours (range: 14 hours to 127 hours). Only healthy newborns whose mothers had no major complications during pregnancy were included in the study. An additional 10 newborns were excluded from the original sample because of fussiness (n = 4), sleepiness (n = 4) or procedural errors (n = 2). The reject decision was decided by the two experimenters. For 13 newborns, parents spoke a language other than French at home.

### Apparatus

Newborns were observed in a quiet room accompanied by one or both parents. Before testing, we systematically ensured that parents and medical staff gave their agreement. Each newborn was positioned in a semi-upright position (30°) in an adapted rigid seat placed on a table facing a 19-inch DELL colour monitor, 35 cm away from the infant's eyes. Two speakers were placed on each side of the monitor. One experimenter (Experimenter 1) stood behind the newborn during the whole session to monitor for potential signs of discomfort. A small video camera was directed at the newborn, recorded the whole experiment (the temporal resolution was 25 images/s), and displayed the images on two video monitors. One monitor allowed a second experimenter (Experimenter 2) to code the duration of looking. The other allowed the parents to see their baby. The parents sat behind and far from the baby, so that the infant could not see them. Parents were instructed to not intervene (speak or come near their baby) during the experiment.

### Stimuli

**Figure 1:**
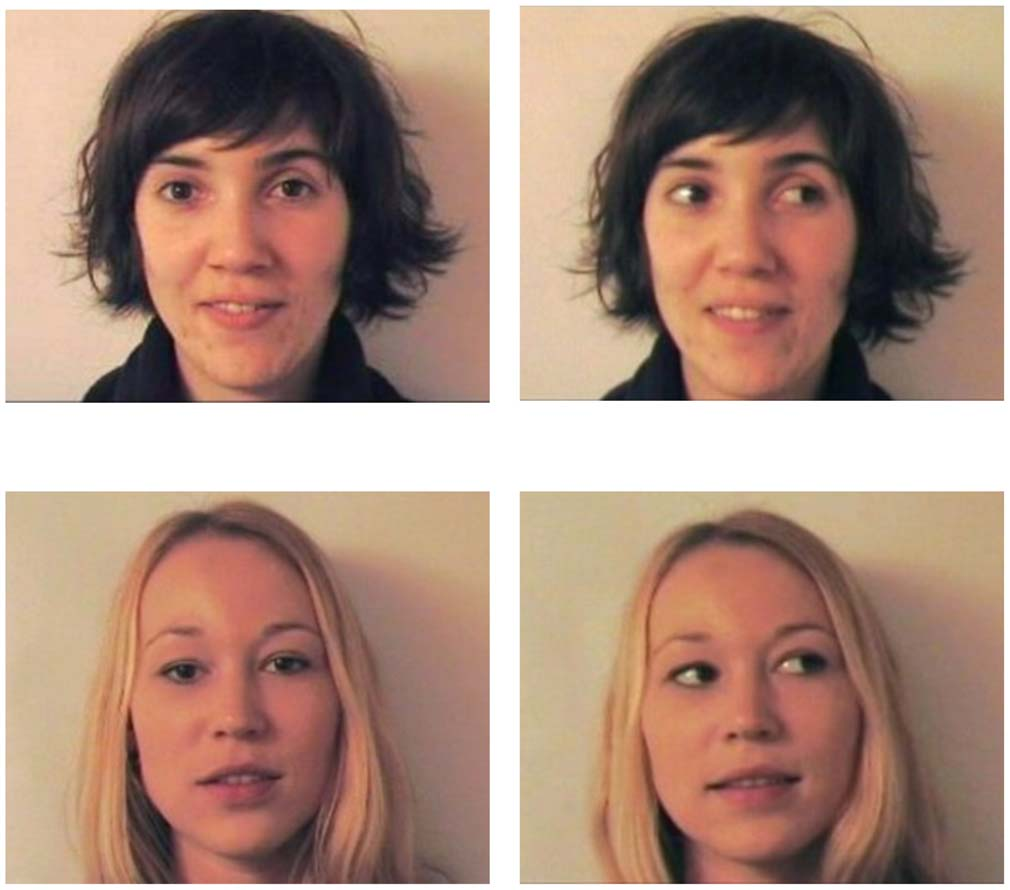
Illustration of the stimuli used in the Experiment: brown and blonde-haired faces with direct or averted gaze.

For the familiarization phase, six different colour video clips of two female faces were recorded. Female faces were used because they are thought to be more attractive than males for young infants and this may maximise attention to the faces during the experiment [7], [8]. These videos were recorded under the same lighting conditions (mean: 16 cd/m^2^) with the same white background in a soundproof room as in Coulon et al. 's study [36]. The two women differed in terms of hair colour and style: short brown hair (brown-haired face) versus long blond hair (blonde face). We chose two different females' faces so that by counterbalancing their presentation across subjects we ensured that results found were not due to physical characteristics of the stimuli. They both previously learned the same text and while video recorded, each woman addressed to the camera in an infant directed speech style with direct or averted gaze (videos can be obtained from the authors on request). In the averted gaze condition, faces were either looking to the right or to the left. Each of the six videos lasted for 80 s. Sound intensities at the speakers in the testing room were identical for all stimuli (mean: 65 dB). For the test phase, the last frame of each familiarization video clip was presented without motion. So, there were three images of the brown-haired face: one with direct gaze, one with right averted gaze, and one with left averted gaze, and same for the blonde face. All facial images in the familiarization and in the test phases were presented at life size (see Figure 1). Each image subtended a visual angle of 40.9×36.1° and the external contour of one eye was approximately 3.3×6.5°.

### Procedure

The experiment began as the infant was seated. The familiarization-test procedure was the same as in Coulon et al. 's experiments [36]. Half of the newborns (n = 12) were tested with the direct gaze condition (i.e., faces presented with direct gaze in the familiarization and in the test phases) and the other half with the averted gaze condition. Moreover, in the averted gaze condition, half of the newborns were presented with right averted gaze and the other half with left averted gaze. The same procedure was applied to all conditions. Newborns were first familiarized with one of the two females' faces talking for 80 s continuously. Half of the newborns were familiarized with the brown-haired face and the other half with the blonde face. Immediately after the familiarization phase, the test phase began. In each of two blocks of test trials, the newborn saw the photograph of the familiar face (i.e., F) and the photograph of the new one (i.e., N) alternatively. Half of the newborns therefore saw the two faces in each order (i.e. FNFN vs. NFNF). A computer program randomly determined which of the four conditions was presented to each of the participants: Familiarization (brown-haired face or blonde face) and Test (FNFN or NFNF).

During the familiarization phase, Experimenter 2, unaware of the face presented, pressed and held a key button on a computer keyboard when the infant looked at the screen and released it when the infant looked away. The computer program recorded the accumulated looking times. During the test phase, Experimenter 2 proceeded in the same way, but when newborns looked away from the screen for more than two seconds, the computer program automatically switched to the next face. A switch also occurred after the newborns had looked at the face for 60 s continuously (i.e., maximum length of each video in the test phase). The computer program also required a minimum of 2 seconds looking time at the screen. Looking times were verified *a posteriori* from the video recordings by Experimenter 1, blind to the experimental conditions. Inter-observer reliability throughout the experiment was high (Pearson's *r* = 0.90, *p*<.01).

## Results

### Familiarization phase

The looking behaviour toward the faces was recorded for each infant as the dependent measure and total looking times were calculated across the two conditions. We tested whether the newborns' attention remained constant during the familiarization phase by comparing the duration of newborns' fixations across the familiarization phase. Newborns looked at the talking faces shown in videos for an equal amount of time, in average, in both conditions (direct gaze: 68.3 s SE = 2.38; averted gaze: 68.1 s SE = 2.62; *t*-test, *p*>.10). In the averted gaze condition, there was no significant difference of mean looking times between right and left averted gaze (right: 62.1 s SE = 3.48; left: 74.1 s SE = 1.96; *t*-test, *p*>.10). Although half of the newborns were familiarized with the brown-haired face and the other half with the blonde face, there was no significant difference in mean looking times between the two faces (brown-haired-face: 65.6 s SE = 2.64; blonde face: 70.8 s SE = 2.09; *t*-test, *p*>.10). There was no significant difference of mean looking time during the familiarization phase between newborn infants whom parents spoke a language other than French at home and newborn infants whom parents spoke only French, in the direct gaze condition (other languages: 69.9 s SE = 4.1; French: 67.2 s SE = 3; *t*-test, *p*>.10) and in the averted gaze condition (other languages: 69.5 s SE = 2.6; French: 65.3 s SE = 6; *t*-test, *p*>.10).

### Test phase

**Figure 2:**
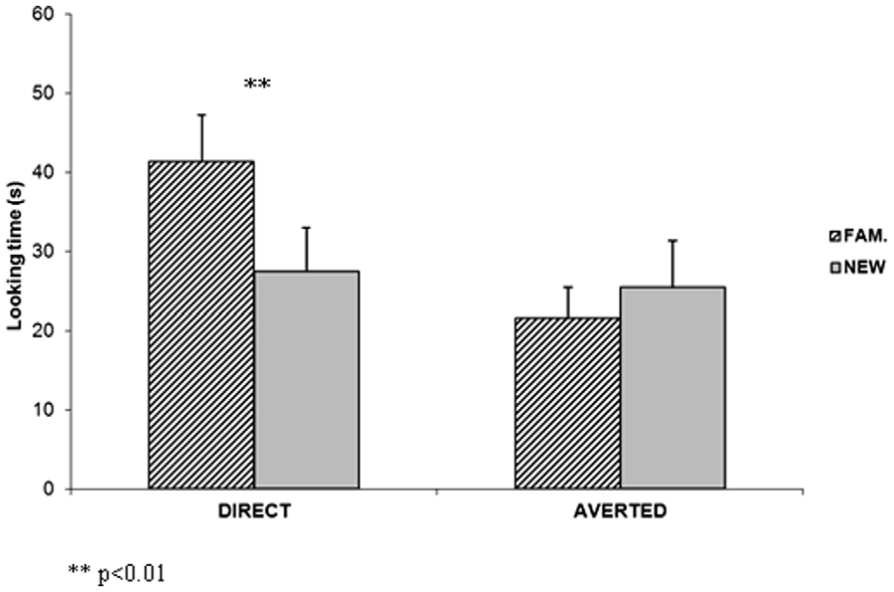
Mean looking time (in seconds) during the test phase at the familiar and at the new faces in both conditions. Error bars represent the standard errors (SE).

During the test phase, mean looking times to the familiar and to the new faces were analyzed in both conditions (see Figure 2). In the direct gaze condition, 10 out of 12 newborns looked longer at the familiar face. Infants looked significantly longer at the familiar face than at the new one (familiar: 41.4 s SE = 5.87; new: 27.4 s SE = 5.53; *t*-test t_(1, 11)_ = 2.4, *p<.01*). In the averted gaze condition, 4 out of 12 newborns looked longer at the familiar face. There was no significant difference in mean looking times between the familiar (21.6 s SE = 3.90) and the new face (25.4 s SE = 5.95; *t*-test t_(1, 11)_ = −1.1, *p>.10*). There was also no significant difference of mean looking time at the familiar and new faces between newborn infants whom parents spoke a language other than French and those whom parents spoke only French, in the direct gaze condition (other languages: familiar  =  49.5 s SE = 11.4, new  =  31.1 s SE = 11.3; French: familiar  =  35.6 s SE = 6.2, new = 24.8 SE = 5.6; *t*-test, *p*>.10) and in the averted gaze condition (other languages: familiar  =  23.7 s SE = 5.7, new = 30.5 s SE = 8.1; French: familiar  =  17.4 SE = 2.3, new =  15.25 SE = 5.5; *t*-test, *p*>.10).

As a previous ANOVA revealed no effect of the order of presentation in each test block: FN or NF, or of the side of averted gaze: right or left, these factors were not taken into account in the final ANOVA. A 2 (Condition: direct or averted gaze) x 2 (Familiarization face: brown-haired or blonde face) X 2 (Block of presentation: 1 and 2) X 2 (Test: brown-haired or blonde face) ANOVA was performed on looking times with the two last factors within subjects. The ANOVA revealed a significant interaction between Condition, Familiarization face and Test (*F*(1, 20) = 6.18; *p*<.02). This interaction confirmed that newborns spent more time looking at the familiar than at the new face, only in the direct gaze condition. No other effect or interaction was significant.

In short, newborns preferred looking at the woman's face that talked to and looked at them simultaneously during the familiarization phase.

## Discussion

The present study aimed at investigating the role of both speech and eye gaze in identifying others at the start of postnatal life. Previous research showed that newborns recognize familiar and unfamiliar persons when these persons had previously interacted verbally with them [33], [36]. Other studies showed that eye gaze is another important social cue that guides newborns' face preference when presented with photographs of unfamiliar faces [49],[50]. By presenting faces in interactive situations, we studied the roles of both cues in guiding newborns' identification of others. The present findings provide evidence that newborns recognize someone who previously talked to them only if this person looked at them directly, and not if their gaze is averted. Using different unfamiliar faces, our findings confirm and extend those already observed in Coulon et al. 's study [36]. In a general manner, newborns recognize and prefer looking at someone who has engaged them in a social interaction by talking to and looking at them simultaneously.

Our findings accord with those of the only research in the domain [47] but with younger infants: eye gaze modulates face recognition not only at 4 months but also soon after birth. This finding is consistent with the hypothesis of an innate module devoted to gaze processing that orients infants to this rich source of social information [1], [51], [52]. When considering research on newborns' sensitivity to eye gaze in face processing, including the present study, different patterns of results appear depending on the nature of the stimuli presented. This disparity of results suggests that the value of the social signal conveyed by the eyes may vary according to the situations presented. In non interactive situations, such as with static images, newborn infants are able to recognize a face presented at different orientation in the familiarization/habituation and in the test phases (full face to ¾ profile or ¾ profile to full face, but not with profile poses) [31]. In the case of ¾ profiles, eye gaze is averted and newborns are still able to recognize the face. This is partly explained by the fact that static ¾ profile presentations of a face promote face recognition of unfamiliar faces by providing with more structural information than full face presentations [53]. Moreover, the social meaning of averted gaze in ¾ profile poses is not the same as in full face. In more complex situations, in face-to-face verbal interactions for example, it is already expected by infants that the person will look at his/her social partner [54] whereas facing someone in ¾ profile will suggest that this person addresses to someone else. Results of the present study are obtained in the context of a face-to-face verbal situation which is more complex than in studies with static faces. Therefore, in this situation, the speech component may drive newborns' attention to the face. Newborn infants' recognition of someone talking to them with averted gaze could be more difficult than processing of static faces with averted gaze as someone talking to them while looking somewhere else is perceived as an incongruent situation. In other words, newborn infants would process faces differently according to the situations presented and would be already sensitive to social congruencies. Moreover, the idea that, at birth, presentation of faces in more complex settings than in previous studies leads to different results' patterns is supported by results of a recent study [32]. In this study, the authors presented to newborns a face displaying a sequential motion of the all head from the left ¾ profile to the right ¾ profile. Then, in the test phase, static profile images of the previously seen face and of a new one were presented simultaneously. In spite of the fact that newborns are unable to recognize a static image of a face from ¾ profile or full face to profile, and vice et versa [31], habituation with more complex stimuli (i.e., faces in sequential rigid motion) enable newborns' recognition of the previously seen face even with profile poses presentations at test.

In static presentations of faces, direct gaze seems sufficient to guide newborns' face processing as it is the main source of social information. When presenting photographs of faces, newborns clearly prefer looking at faces with direct gaze [49],[50] whereas in interactive situations, for example in front of silent or talking moving faces, newborns exhibit a preference only for a face that has interacted with them verbally [36]. In this case, direct gaze alone is not a sufficient cue for newborns' identification of others in the absence of speech. It is only around 3 months of age that this cue will be understood by infants with a different meaning such as indicating the presence of objects [55]. In this context, infants will develop gaze following in response to others' gaze, and interacting with others can be considered as being still the main motive of this behaviour [56].

In the other hand, the present findings show that the presence of infant-directed speech is necessary but not sufficient: simultaneous direct gaze is required as well. In daily situations, newborns see various unfamiliar faces, moving and/or talking. It is possible that according to the situation, newborns have different expectations that need more specific cues to identify others as potential social partners. Speech and direct gaze seem particularly useful in this process. This finding accords with the hypothesis of a core system for representing potential social partners as suggested by some authors [5], [6]. Such system would orient infants from birth toward persons identified as interesting social partners and would help in the construction of social bonds. However, in contrast to their studies, no effect of the maternal language was found in our experiment. Newborns were sensitive to someone who interacted with them verbally *per se* no matter if the person spoke a different language from the one heard in the family. This finding suggests that at birth, infants have a bias for speech in its socio-communicative dimension, as do other species [57]. Perhaps, later in the middle of the year after birth, infants' social preferences start being clearly influenced by their social environment. In this process, their native language becomes a major cue in establishing social categorizations and preferences as revealed by a previous study in 6-month-old infants [22]. However, it is also plausible that native language is a major cue at birth. To disentangle between these two hypotheses, the same experiment as that with 6-month-olds [22] should be realized at birth.

Taking together the present findings and those of previous research on the role of socio-communicative cues in guiding infants' identification of others, a developmental line can be drawn. At birth and also at 1 month, situations of interaction and more precisely of verbal interaction are necessary in guiding young infants' identification of familiar [33], [37] and unfamiliar persons [36]. In these situations, direct gaze is important and modulates newborns' identification of the person who talked to them. At 3 and 5 months, such situations of verbal interactions are not strictly necessary in guiding infants' identification of their mother's face as they are able to recognize her even if she is seen with static lips associated speech sounds [37].

In sum, the present findings suggest that at birth, infants are already able to identify others by means of two socially meaningful cues, and that interactive situations are privileged in eliciting preferences for potential social partners. To understand more precisely the mechanisms underlying the construction of early social interactions and to confirm the possible existence of a system dedicated to representations of potential partners from birth, further investigations are needed. For example, still in the situation of verbal interactions, the importance of other cues could be investigated such as speech prosody, known to have particular characteristics in infant directed speech and which function is highly social as it may drive language acquisition [58], [59].
